# Language as a catalyst for success: a blueprint for standardized terminology and multilingual health literacy in Pakistan

**DOI:** 10.1016/j.lansea.2025.100544

**Published:** 2025-02-14

**Authors:** Humza Irfan, Areeb Farooq, Mohammad Usama Toseef

**Affiliations:** a College of Literature, Sciences, and the Arts, University of Michigan, United States of America; b Department of Biological Sciences, Fairleigh Dickinson University, United States of America; c Corewell Health, United States of America

Pakistan's rich linguistic diversity, with over 70 languages spoken and 14 recognized as major languages, presents a critical opportunity to enhance healthcare delivery and promote health equity across the country.^1^^,^^2^ Especially in rural areas, where local languages dominate, there is an urgent need for healthcare providers, who are predominantly trained in English, to incorporate regional languages into their communication practices. Addressing this promptly can bridge existing communication gaps, foster trust, and strengthen patient–provider relationships.^3^ A study in a tertiary care hospital revealed that 68.6% of patients were not consulted on the quality of their care.^4^ These statistics in-part reflect the systemic failure to bridge linguistic gaps, with repercussions including misdiagnoses, inappropriate treatments, and diminished adherence to medical advice. The psychological toll on patients, often overlooked, exacerbates anxiety and alienation, further eroding trust in healthcare providers^5^ (Fig. 1).

**Fig. 1:**
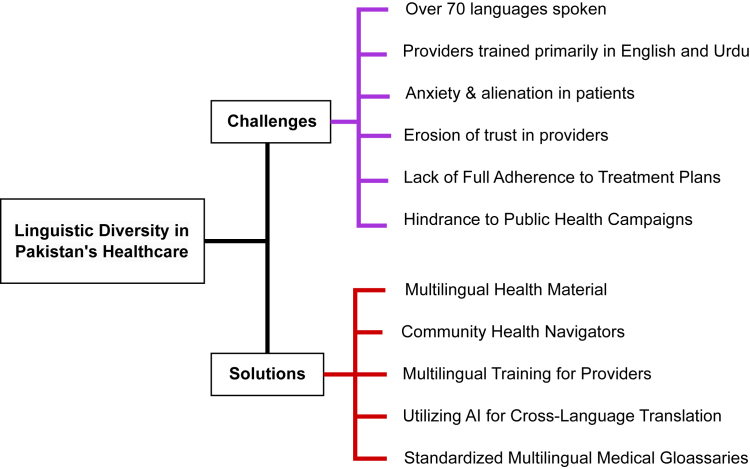
A visual representation of the challenges and solutions related to linguistic diversity in Pakistan's healthcare system.

Efforts to address these challenges must be multifaceted. A key strategy involves developing multilingual health education materials tailored to local contexts. These resources can bridge communication gaps, particularly in preventive care and health literacy campaigns.^6^ Public health messages, whether about vaccination drives, maternal health, or disease prevention, must be communicated in languages understood by the target audience. The success of these campaigns depends on their ability to effectively engage and inform the population.

For example, during the COVID-19 pandemic, disseminating information in local languages was critical to ensuring that all segments of the population received accurate information about the virus and the importance of vaccination. However, the lack of standardized health communication in local languages often led to misinformation and vaccine hesitancy.^7^

Engaging community health navigators as cultural brokers and language interpreters is essential for improving access to healthcare services. Some regions may have relatively more linguistic uniformity, while others display a more complex linguistic landscape. Recognizing these differences is crucial for tailoring language-based interventions. Such efforts can enhance the effectiveness of public health campaigns and ensure that health messages are clearly understood and culturally appropriate for diverse communities.^8^

Furthermore, integrating language and cultural competency training into healthcare curricula is essential for preparing future providers to communicate effectively with diverse patient populations. Communication is a cornerstone of quality healthcare, and training health professionals adequately can help reduce health disparities and promote health equity. Practical components, such as internships and community service projects, can further enhance this training by providing hands-on experience that equips providers to navigate linguistic and cultural nuances in real-world patient care.^9^

AI-driven tools hold promise for reaching diverse language communities, rapidly converting medical instructions, public health messages, and patient-provider interactions into multiple languages.^10^ Yet, limited infrastructure, poor connectivity, and low computer literacy hinder widespread adoption in rural Pakistan. Addressing these barriers is crucial for realizing AI's potential to improve accessibility and accuracy in patient-provider communication.

Lastly, the most critical step is the development and standardization of healthcare terminology across Pakistan's major languages. This involves creating comprehensive glossaries of terms that are culturally and linguistically appropriate for various regions and accepted for use in healthcare settings. The absence of standardized medical glossaries in Pakistan's languages leads to significant semantic and conceptual ambiguities. For providers trained in English, communicating health terminology in different languages becomes a challenge, as there are no standardized equivalents that convey a unified meaning.

Furthermore, even terms that might have translations may not convey the same meaning. For example, terms like “hypertension” or “immunity” often lack precise equivalents in local languages, leading to oversimplifications. “Hypertension” might be broadly translated as “high blood pressure,” failing to distinguish between pre-hypertension, stage 1, or stage 2 hypertension.^11^ Similarly, translations of “immunity” may fail to fully capture its scientific nuances, potentially leading to misconceptions.

These discrepancies can result in critical misinterpretations in clinical settings, where patients may misunderstand the severity of their condition or where providers may inadvertently rely on imprecise terms that hinder accurate diagnosis and treatment planning. Collaboration between linguists, healthcare professionals, and government agencies is essential to ensure the accuracy and broad acceptance of medical terminology in Pakistan's languages. The creation of standardized glossaries is the first step in addressing these issues, and regular updates are necessary to reflect advancements in medical knowledge and changes in language use. Such efforts can help reduce ambiguities in communication, particularly in regions where technical terms may not have direct equivalents in local languages.^12^

Clear, inclusive communication is fundamental to equitable healthcare, and Pakistan must prioritize linguistic inclusivity to improve health outcomes. The stakes are high: without urgent action, the country risks perpetuating health inequities that disproportionately affect its most vulnerable populations. With the right strategies, however, Pakistan can leverage its linguistic richness to promote greater health equity and better serve its diverse populations.

## Contributors

HI conceptualized, outlined, researched, and prepared multiple drafts, including the final draft. AF contributed to source research and prepared the initial draft. MUT provided edits and reviewed the manuscript.

## Declaration of interests

None declared.
